# Absence of the proteoglycan decorin reduces glucose tolerance in overfed male mice

**DOI:** 10.1038/s41598-018-37501-x

**Published:** 2019-03-15

**Authors:** Jessica Svärd, Therese H. Røst, Camilla E. N. Sommervoll, Christine Haugen, Oddrun A. Gudbrandsen, Anne E. Mellgren, Eyvind Rødahl, Johan Fernø, Simon N. Dankel, Jørn V. Sagen, Gunnar Mellgren

**Affiliations:** 1 KG Jebsen Center for Diabetes Research, Department of Clinical Science, University of Bergen, N-5020 Bergen, Norway; 2 Hormone Laboratory, Haukeland University Hospital, N-5021 Bergen, Norway; 3 Department of Clinical Medicine, University of Bergen, N-5020 Bergen, Norway; 4 Department of Ophthalmology, Haukeland University Hospital, N-5021 Bergen, Norway

## Abstract

Studies have implicated the extracellular matrix (ECM) of adipose tissue in insulin resistance. The proteoglycan decorin, a component of ECM, has been associated with glucose tolerance, but possible causal effects on metabolism remain to be explored. We here sought to determine metabolic consequences of loss of decorin in mice (*Dcn*KO). *Dcn*KO mice were fed a low-fat (LF) or high-fat (HF) diet for 10 weeks and body weight and food intake was recorded. An intraperitoneal glucose tolerance test was performed after eight weeks. Blood samples and adipose, liver and muscle tissues were collected at sacrifice. Global gene expression was measured in adipose tissue, and expression of decorin was also analyzed in human adipose samples. *Dcn*KO mice showed increased feed efficiency during overfeeding and impaired glucose tolerance. Adipose leptin mRNA and circulating leptin levels were elevated in *Dcn*KO mice, along with a downregulation of genes involved in ECM organization and triglyceride biosynthesis, and an upregulation of adipose genes involved in complement and coagulation cascades. Consistent with a protective metabolic role for decorin, in obese patients we found increased adipose decorin expression after profound fat loss, particularly in the stromal vascular fraction. Loss of decorin in mice caused impaired glucose tolerance in association with increased feed efficiency and altered gene expression in adipose tissue. Our data provide evidence that decorin is an important factor for maintaining glucose tolerance.

## Introduction

Obesity has become more prevalent during the last decades, and it is associated with metabolic disturbances which can be related to structural changes in tissues and cross-talk between different organs. During development of obesity, gene programs are activated within adipose tissue to support tissue expansion^1,2^. Adipose tissue is a loose connective tissue where adipocytes and adipocyte precursors are embedded in an extracellular matrix (ECM) composed of structural proteins (e.g., collagens and elastin) and adhesion proteins (e.g., proteoglycans), providing structural support and also regulating intercellular communication^3,4^. Synthesis and degradation of ECM proteins may play an important role in the regulation of adipose tissue size and function during obesity progression^5^, and expression of ECM proteins and ECM remodeling pathway are altered in obesity and type 2-diabetes^6^. Several ECM components, including collagen type VI, biglycan and tenascin C, have been implicated in metabolic dysregulation in obesity^7–13^.

The small leucine rich proteoglycan decorin (Dcn) is a component of the ECM in many tissues. It is highly expressed in adipose tissue, and more highly expressed in visceral compared to subcutaneous adipose tissue depots^14,15^. Experiments with fractionized adipose tissue show that expression of decorin is highest in the non-adipocyte stromal vascular fraction (SVF) that contains, e.g., adipocyte precursors^14^. Previous studies of rodents and humans with obesity and glucose intolerance have suggested increased expression of decorin mRNA in whole adipose tissue and secreted decorin protein in plasma^14,16^. Leptin may be involved in the regulation of decorin expression since ob/ob mice, despite a massive increase in body weight, have a reduced expression of decorin in epididymal adipose tissue^16^. Furthermore, decorin polymorphisms were recently found to associate with higher serum glucose in humans^17^. Decorin may at least partly influence metabolism and adipose tissue expansion through its function as a receptor on adipocyte progenitors for the adipokine resistin^18^, and SNPs near the decorin (*DCN*) gene were found to associate with circulating resistin in Japanese people^19^. Furthermore, decorin interacts with several molecules present in the ECM, including structural proteins such as types VI, XII and XIV collagen, fibronectin and elastin^20–24^, and growth factors such as EGF, TGFβ, TNFα and myostatin^25–28^. These interactions may, in addition to stabilizing the extracellular matrix, also participate in regulating the metabolism^29^ and activity of these growth factors at its receptors. These findings indicate that decorin may play an important role in adipose tissue function and in the pathophysiology of obesity.

In the present study, we used the well-established C57BL/6 mouse model for obesity and impaired glucose tolerance and type 2 diabetes^30^, to test a possible causal impact of decorin on body weight, glucose tolerance and adipose tissue in high-fat challenged *Dcn* null (*dcn*KO) C57BL/6J mice.

## Materials and Methods

### Animals and diets

**Figure 1:**
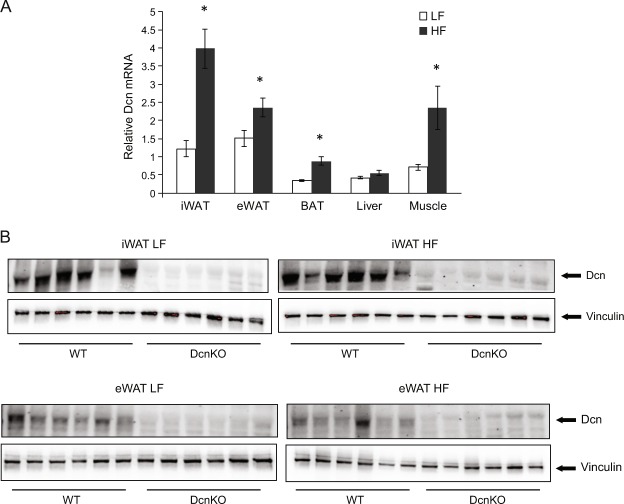
*Dcn* mRNA and protein expression in different metabolic tissues of *Dcn*KO and wild-type (WT) C57BL/6J mice. Male mice were fed a low-fat (LF, n = 8) or high-fat (HF, n = 10) diet for 10 weeks, and tissues were collected immediately after being euthanized. RNA was purified, cDNA was synthesized and relative gene expression was measured by qPCR using *Rps13* as a reference gene. iWAT, inguinal white adipose tissue; eWAT, epididymal white adipose tissue; BAT, interscapular brown adipose tissue. *p-value < 0.05. (**A**) Protein levels of Dcn in different adipose tissues was determined by western blotting relative to the reference Vinculin (**B**).

The animal experiment was performed in accordance with the Norwegian regulation on animal experimentation. The protocol was approved by the Norwegian State Board of Biological Experiments with Living Animals (Approval No. 2013–6770). The male mice were kept at the Vivarium, University of Bergen, Norway. The *Dcn* knock-out strain was created by the use of homologous recombination, which was performed by genOway, Lyon, France. Removal of exon 8 by mating with Cre-expressing mice resulted in degradation of *Dcn* mRNA. No decorin protein could be detected by western blot in the *Dcn* KO strain^31^ and Fig. 1B. The strain was back-crossed for 9 generations using C57BL/6J (BomTac) before experiments and is available at The Jackson Laboratory Repository with the JAX Stock No. 27672, B6.129S(Cg)-*Dcn*^*tm1*.*2Geno*^/AecmJ (http://jaxmice.jax.org/query). C57BL/6J (BomTac) (WT) mice or *Dcn*KO on the same background were housed, one per cage, at constant temperature (22 ± 2 °C) and humidity (55 ± 5%), and exposed to a 12 h light–dark cycle with unrestricted access to food and tap water.

Eight male WT mice and eight male *Dcn*KO mice were fed a low fat diet (LF) with 3,85 kcal/g, 10 kcal% fat, 70 kcal% carbohydrates, 20 kcal% protein and 35 kcal% sucrose (Research Diets, New Brunswick, NJ; Cat. No. D12450B) and 10 WT mice and 10 *Dcn*KO mice were fed a high fat diet (HF) with 4,73 kcal/g, 45 kcal% fat, 35 kcal% carbohydrates, 20 kcal% protein and 17 kcal% sucrose (Research Diets, New Brunswick, NJ; Cat. No. D12451) for 10 weeks starting at an age of 12 weeks. Diets were packed airtight and stored at 4 °C until used to prevent lipid oxidation. Body weights were measured once each week.

### Food intake

The weights of food given and remaining in cages were measured once each week and the consumed total amount of food was calculated.

### RNA isolation and qPCR analysis

Total RNA was extracted from 10–100 mg of tissue using the RNA/Protein Purification Kit (product 24100, Norgen Biotek Corporation, Canada) following the manufacturer protocols. The concentration of RNA was determined by spectrophotometry (Nanodrop ND-1000; NanoDrop Technologies, Wilmington, DE), and RNA integrity was confirmed by bioanalysis (Agilent 2100 bioanalyzer; Agilent Technologies, Inc., Santa Clara, CA).

RNA samples (0.5 μg) were reverse transcribed using the Applied Biosystems™ High-Capacity cDNA Reverse Transcription Kit. The cDNA template was synthesized following the protocol that includes 10 uL RNA sample, 2.0 uL 10X RT Buffer, 0.8 uL dNTP Mix, 2.0 uL10X RT Random Primers, 1.0 uL MultiScribe™ Reverse Transcriptase, 1.0 uL RNase Inhibitor, and 3.2 uL nuclease-free H_2_O. Total volume per reaction was 20.0 uL.

Genes of interest were analyzed by individual real-time SYBR green PCR assays with HPRT or RPS13 used as an internal control. The SYBR Green based PCR was carried out on the LightCycler® 480 Real-Time PCR System (Roche). The PCR reaction was prepared in a total volume of 10 uL containing 1.5 ul cDNA template, 0.5 ul of each primer (20 uM), 5 ul of SYBR Green and 2.75 deionized water. A negative control was established by replacing cDNA template with deionized water. The expression level of each gene was calculated relative to the expression of the reference gene using the 2^−*ΔΔCt*^ method.

### Protein isolation and western blot analysis

Protein was isolated from tissues using the RNA/Protein Purificatiion Plus kit (Cat. 48200); Norgen Biotek. Protein samples (10 µg total protein) were separated by electrophoresis in a 10% SDS-polyacrylamide gel (TGX Precast Protein Gels) and subsequently transferred to a nitrocellulose membrane (Nitrocellulose Transfer Pack) using the Trans-Blot® Turbo Transfer System (Bio-Rad Laboratories, Inc.). All membranes were transferred by the 7 minute pre-set setting and blocked for 1 hour at room temperature (RT) in 5% non-fat dry milk in Tris-buffered saline (TBS; 20 mm Tris-HCl, 140 mM NaCl pH 7.4), containing 0.1% Tween® 20 (0.1% TBS-T). Immunodetection was carried out by incubating the primary antibody of interest at 4 °C overnight in either 3% BSA (α-hDecorin (1:2000); AF143 *R&D Systems* and α-pAKT, Ser473, (1:1000); *Cell Signaling Tech*.*®*) or 5% BSA (α-Vinculin (1:2000); *Abcam* and α-AKT (1:1000); *Cell Signaling Tech*.*®*). HRP-conjugated goat-anti-rabbit IgG or goat-anti-rabbit IgG was used as the respective secondary antibodies (*Cell Signaling Tech*.*®*). Between all steps throughout the procedure, membranes were washed extensively in 0.1% TBS-T at RT. Anti-Vinculin was verified by comparing signals to stain-free total protein measurements and subsequently used as loading control in all immunoblots. Chemiluminescence of secondary antibody-HRP conjugates was elicited using SuperSignal West Femto Maximum Sensitivity Substrate (Thermo Scientific) and imaged with Gel DocTM XR+ (Bio-Rad Laboratories, Inc.) and quantified using Image Lab 6.0 software (Bio-Rad Laboratories, Inc.).

### Blood and tissue samples

Animals were fasted for 6 hours, anesthetized with 2% isoflurane (Schering-Plough, Kent, UK) and blood was collected by heart puncture before the mouse was euthanized. The blood was centrifuged, EDTA-plasma separated and stored at −80 °C prior to further analysis. Liver, epididymal white adipose tissue (eWAT), inguinal WAT (iWAT), skeletal muscle (*gastrocnemius*) and BAT were collected and immediately frozen in liquid nitrogen and stored at −80 °C until further analysis.

Fasting EDTA-plasma samples at study endpoint were analyzed for Total Cholesterol, HDL Cholesterol, LDL Cholesterol, Triglycerides, Non-esterified fatty acids (NEFA) using the Cobas C111 System (Roche Diagnostics GmbH, Mannheim, Germany). Standard kits were used for all except for NEFAs that were analyzed using the NEFA FS kit (DiaSys, Diagnostic Systems GmbH, Germany). Lipids were measured by enzymatic colorimetric assays with specific reagents from Roche Diagnostics for total cholesterol (CHOL2, Cat. No. 04718917190), triglycerides (TRIGL, Cat. No. 04657594190), HDL cholesterol (HDL, Cat. No. 05401488190), and LDL cholesterol (LDL C, Cat. No. 04657578190). Insulin (Cat. No. 90080) and Leptin (Cat. No. 90030) were measured by ELISA from (Crystal Chem, Downers Grove, IL, USA).

### Glucose tolerance test (GTT)

Intraperitoneal (IP) GTTs were performed at 20 weeks of age (after 8 weeks on HF or low fat diet (LF)). For GTTs animals were fasted for 5 hours, and glucose (2 g/kg body weight) was injected IP. Blood glucose was measured from the saphenous vein using a glucometer (Contour, Bayer) at 0, 15, 30, 60, 120 and 180 min after glucose injection in un-anesthetized mice.

### Histology and adipocyte size measurement

Formalin fixed, paraffin embedded eWAT was sectioned (5 μm) on a Leica RM2255 microtome. Hematoxylin and eosin (H&E) staining was performed on three to five sections per animal. Images were captured under bright-field illumination using a Nikon microscope (E800; Tokyo, Japan) with a Plan Apo ×10 (NA 0.75) objective.

### Microarray analysis

Global gene expression analysis was performed in iWAT from 8 WT and 8 *Dcn*KO mice on LFD or HFD using Illumina Mouse Microarray at The Genomics Core Facility (GCF) at the University of Bergen.

### Patients

A description of human patients can be found in^32^. The study of human subjects was approved by the Regional committee for medical and health research ethics, western Norway (REC West). Each subject gave written informed consent, and the study was performed in accordance with the declaration of Helsinki.

### Statistical analyses

Statistical analyses were conducted using SPSS Statistics 22. All values are represented as the mean ± sem. Significance between two groups was assessed using 2-way ANOVAs or two-tailed unpaired or paired Student’s *t*-test. *P* < 0.05 was considered significant. Number of animals and persons used for all measures are provided in figure legends.

## Results

### Diet-induced increase in *Dcn* mRNA expression in different adipose tissues

Studies have shown that rodents and humans with obesity and glucose intolerance have increased expression of decorin in adipose tissue^14,16^. In this study we further examined diet-dependent decorin expression in different adipose tissue depots and other metabolic tissues of mice fed a control low-fat diet. As expected we found that high-fat (HF) feeding increased *Dcn* mRNA in epididymal white adipose tissue (eWAT) (Fig. 1A). We also found increased expression of *Dcn* in inguinal white adipose tissue (iWAT), brown adipose tissue (BAT), and skeletal muscle, whereas there was no difference in the hepatic gene expression (Fig. 1A). To confirm loss of Dcn protein in tissues of *Dcn*KO mice, we purified protein from iWAT and eWAT of WT and DcnKO mice fed a LF or a HF diet. We could not detect expression of Dcn in inguinal white adipose tissue (iWAT) or epididymal white adipose tissue (eWAT) of *Dcn*KO mice. The reference gene, Vinculin, was detected as a technical control (Fig. 1B).

### Increased weight gain and feed efficiency in *Dcn*KO

**Figure 2:**
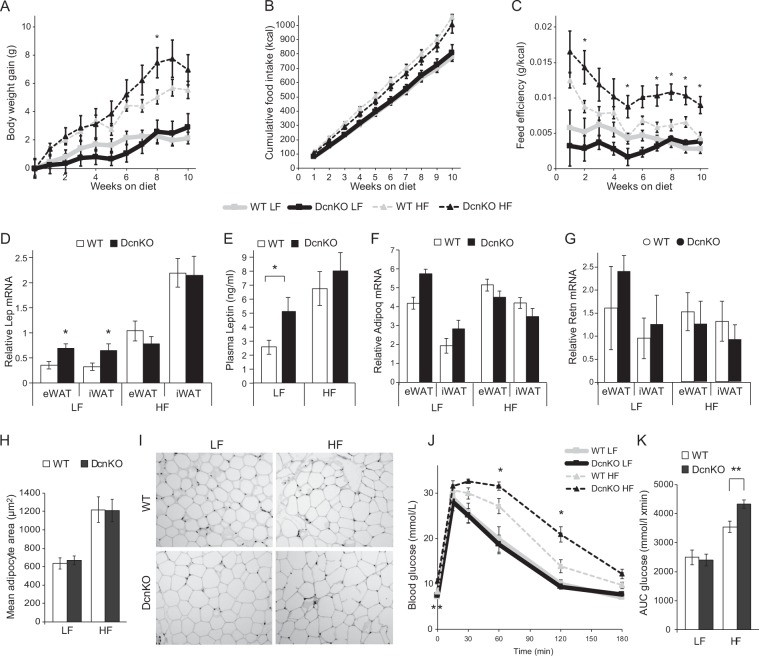
Changes in metabolic phenotype in *Dcn* knock-out (*Dcn*KO) relative to wild-type (WT) C57BL/6J mice. Twelve week old male mice were subjected to a low-fat (LF) or high-fat (HF) diet for 10 weeks. Body weight (**A**) and food intake (**B**) were measured every week, and feed efficiency (grams of weight gain per unit of caloric intake) was calculated (**C**). *Lep* mRNA in adipose tissue was measured by qPCR calculated relative to the reference gene Rps13 (**D**), and circulating levels of leptin were measured in plasma by ELISA (**E**). *Adipoq* and *Retn* mRNA in adipose tissue was measured by qPCR calculated relative to the reference gene Rps13 (**F**,**G**). Mean adipocyte size in inguinal white adipose tissue (iWAT) was calculated by measuring 50–100 adipocytes on 3–5 slides per animal (**H**). Representative images of the hematoxylin and eosin (H&E) stained adipose tissue are shown (**I**). A glucose tolerance test (GTT) was performed after 8 weeks on the diets, with intraperitoneal glucose injection (2 g/kg body weight) after a 5 hour fast (**J**), and area under the curve (AUC) was measured based on the repeated measurements of blood glucose (**K**). *p-value < 0.05, **p-value < 0.01.

To test the role of decorin in body weight regulation, we fed WT and *Dcn*KO mice a HF or LF diet. At study start the body weights were not different in the LF groups 25.2 ± 0.6 g (WT) and 26.1 ± 0.7 g (*Dcn*KO), or in the HF groups 27.5 ± 0.3 g (WT) and 26.7 ± 0.7 g (*Dcn*KO) respectively. Both WT and KO mice gained considerably more weight on HF compared to LF diet (Fig. 2A). During the first four weeks, the WT and *Dcn*KO mice fed HF gained similar amounts of weight. In contrast, the *Dcn*KO mice gained surprisingly more weight, with a significant difference in accumulated weight gain seen at 8 weeks (Fig. 2A). However, after the full 10 weeks on HF, weight gain did not differ significantly between the two genotypes (Fig. 2A), which was also the case on LF (Fig. 2A). Moreover, we did not find an interaction between diet and genotype calculated by a 2-way ANOVA. However, while food intake was not different between the genotypes on either HF or LF diet (Fig. 2B), the *Dcn*KO mice showed significantly higher feed efficiency (amount of weight gained relative to food intake) compared to WT on the HF diet (week 10, Fig. 2C). Feed efficiency was not significantly different between the two genotypes after 10 weeks of LF feeding (Fig. 2C).

### Higher concentration of leptin in *Dcn*KO mice

The difference in body weight and feed efficiency between WT and KO mice upon HF feeding prompted us to measure circulating leptin, which reflects fat mass^33^. As expected, HF feeding increased the levels of leptin both in plasma and in iWAT and eWAT compared to LF (Fig. 2D,E). Interestingly, plasma leptin as well as mRNA levels of leptin in eWAT and iWAT were higher in *Dcn*KO than in WT fed a LF diet, but the higher level of leptin upon HF feeding in both genotypes gave no significant difference during HF (Fig. 2D,E). Moreover, we found no significant differences in the expression of adiponectin or resistin mRNA in iWAT or eWAT between WT and *Dcn*KO animals (Fig. 2F,G).

Leptin has previously been shown to associate more closely with adipocyte hypertrophy than hyperplasia^34^, and we therefore further assessed adipocyte size in eWAT of the *Dcn*KO and WT mice. In agreement with the higher leptin upon HF compared to LF feeding, adipocytes in eWAT were larger in mice after the HF diet for both genotypes (Fig. 2H,I). However, we observed no significant differences between the genotypes after either diet (Fig. 2H,I), suggesting that adipocytes in the *Dcn*KO mice were unable to expand in size beyond the diet effect. Because leptin has a proadipogenic (i.e. hyperplasic) effect on preadipocytes^35^, the higher leptin upon LF feeding in the *Dcn*KO mice may reflect an effect of decorin on leptin-mediated adipogenesis/hyperplasia, rather than primarily on hypertrophy.

### Reduced glucose tolerance in *Dcn*KO mice on HF

Changes in the extracellular matrix have been demonstrated to play a causal role in fat mass regulation as well as in whole-body glucose tolerance^8^. Accordingly, we wanted to find out whether *Dcn*KO mice would show altered glucose metabolism in response to HF feeding. After 8 weeks on HF, the fasting glucose level was significantly higher in *Dcn*KO mice (10.6 ± 0.6 mmol/L) compared to WT mice (8.3 ± 0.4 mmol/L) (Fig. 2J). Further, we performed an intraperitoneal glucose tolerance test (GTT), revealing higher glucose levels at 60 and 120 min in *Dcn*KO mice given HF feeding compared to WT mice on the same diet. This translated into a significantly higher area under the curve (AUC) glucose for *Dcn*KO compared to WT mice (Fig. 2K). No difference in glucose tolerance was found between WT and *Dcn*KO mice on LF. No significant differences in plasma insulin concentrations were observed between the groups, even though it was higher in *DcnKO* mice in both the LF and HF groups (Table 1), suggesting that *Dcn*KO mice had some increase in insulin resistance compared to WT animals. The difference in glucose levels could not be explained by differences in the expression levels of different glucose transporters or any apparent differences in insulin signaling (Fig. S1). However, we found a small but significantly decreased expression of Ucp1 in BAT from *DcnKO* mice fed HF (Fig. S2).

**Table 1:** Biochemical parameters in plasma of wt and *Dcn*KO mice.

	LF		HF	
	Wt	*Dcn*KO	Wt	*Dcn*KO
Cholesterol (mmol/L)	2,14 ± 0,07	2,05 ± 0,14	2,87 ± 0,25	2,50 ± 0,07
HDL (mmol/L)	2,01 ± 0,04	1,94 ± 0,13	2,76 ± 0,27	2,45 ± 0,10
LDL (mmol/L)	0,41 ± 0,06	0,30 ± 0,02	0,39 ± 0,07	0,22 ± 0,04
TG (mmol/L)	0,45 ± 0,03	0,58 ± 0,04*^#^	0,58 ± 0,03	0,69 ± 0,04
NEFA (mmol/L)	0,24 ± 0,03	0,28 ± 0,02	0,23 ± 0,03	0,24 ± 0,06
Insulin (ng/ml)	0,40 ± 0,08	0,64 ± 0,14	0,62 ± 0,14	0,87 ± 0,24
Bile acids (mmol/L)	6,2 ± 2,3	4,3 ± 1,5	2,6 ± 1,1	3,8 ± 0,4

LF, low-fat; HF, high-fat; HDL, high-density lipoprotein; LDL, low-density lipoprotein; TG, triglycerides; NEFA, Non-esterified fatty acids. #Significance level for comparison of Wt mice with *Dcn*KO mice.

### Effect of genotype and diet on lipids

To gain further insight into the metabolic consequences of loss of decorin circulating lipids were analyzed (Table 1). Plasma concentrations of total cholesterol, HDL cholesterol, LDL cholesterol, Non-Esterified Fatty Acids (NEFA) and total bile acids were not different between the WT and *Dcn*KO groups on either diet. However, the triglyceride concentration was significantly higher in the *Dcn*KO group on the LF diet, and a similar tendency was seen for the HF diet.

### Differentially expressed genes in adipose tissue

To evaluate possible mechanisms involved in the lower glucose tolerance in the *Dcn*KO mice, we measured global gene expression in iWAT from the WT and *Dcn*KO mice after 10 weeks on either diet. For the LF diet, we found 10 transcripts with higher expression and 10 transcripts with lower expression in *Dcn*KO compared to WT, within a false discovery rate (FDR) cut-off of 0 (Table 2). With the same cutoff on the HF diet, we found 22 transcripts with higher expression and 20 transcripts with lower expression in *Dcn*KO compared to WT. Decorin was confirmed to be the most downregulated gene after both diets, and *Prtn3* was one of the most downregulated and *Hal* the most upregulated gene in *Dcn*KO compared to WT mice on both diets (Tables 2 and 3). Prtn3 (proteinase 3) is a serine protease that degrades elastin, fibronectin, laminin, vitronectin, and collagen types I, III, and IV^36^, whereas Hal (Histidine ammonia-lyase) catalyzes breakdown of histidine, an amino acid shown to improve insulin resistance in obese women^37^.

**Table 2:** Differentially expressed genes in adipose tissue of low fat fed mice.

Probe-ID	Gene symbol	Full Gene Name	Fold Change
ILMN_2747959	Dcn	Decorin	−74.85
ILMN_2596346	Dcn	Decorin	−6.10
ILMN_2666864	Atp2a1	ATPase Sarcoplasmic/Endoplasmic Reticulum Ca2+ Transporting 1	−6.44
ILMN_2758029	Prtn3	Proteinase 3	−2.82
ILMN_2481133	Tnni2	Troponin I2, Fast Skeletal Type	−5.35
ILMN_1218223	Pvalb	Parvalbumin	−4.49
ILMN_2977331	Mylpf	Myosin Light Chain, Phosphorylatable, Fast Skeletal Muscle	−3.92
ILMN_2882658	Tnnc2	Troponin C2, Fast Skeletal Type	−4.08
ILMN_2482209	Tpm2	Tropomyosin 2 (Beta)	−3.33
ILMN_2469018	Tnnt3	Troponin T3, Fast Skeletal Type	−3.00
ILMN_2875730	Mup1	Major urinary protein 1	2.38
ILMN_1213817	Mup3	Major urinary protein 3	3.55
ILMN_2443330	Ttr	Transthyretin	3.39
ILMN_2623393	Apoa1	Apolipoprotein A1	2.62
ILMN_2788223	Kng1	Kininogen 1	1.96
ILMN_2659680	Serpina1b	Serpin Family A Member 1	2.38
ILMN_1247156	Apoa2	Apolipoprotein A2	4.01
ILMN_2614752	Elovl6	ELOVL Fatty Acid Elongase 6	2.38
ILMN_1225730	Fdps	Farnesyl Diphosphate Synthase	1.82
ILMN_2984332	Hal	Histidine Ammonia-Lyase	3.31

**Table 3:** Differentially expressed genes in adipose tissue of high fat fed mice.

Probe-ID	Gene symbol	Full Gene Name	Fold Change
ILMN_2747959	Dcn	Decorin	−168.9
ILMN_2596346	Dcn	Decorin	−7.61
ILMN_2758029	Prtn3	Proteinase 3	−2.57
ILMN_2661366	Tmem45b	Transmembrane Protein 45B	−2.18
ILMN_3105563	Dmkn	Dermokine	−1.31
ILMN_1229763	Dmkn	Dermokine	−1.34
ILMN_1232524	Hist1h4i	Histone Cluster 1 H4 Family Member I	−1.68
ILMN_3125966	Kcnj15	Potassium Voltage-Gated Channel Subfamily J Member 15	−1.61
ILMN_2798129	C6	Complement C6	−1.67
ILMN_2710905	S100a8	S100 Calcium Binding Protein A8	−1.60
ILMN_2868152	Krtdap	Keratinocyte Differentiation Associated Protein	−1.30
ILMN_1216720	C6	Complement C6	−1.55
ILMN_1256775	Thrsp	Thyroid Hormone Responsive	−1.50
ILMN_1220275	Nrg4	Neuregulin 4	−1.48
ILMN_2863532	Lipf	Lipase F, Gastric Type	−1.43
ILMN_2948296	Wfdc12	WAP Four-Disulfide Core Domain 12	−1.41
ILMN_2870696	Hfe	Hemochromatosis	−1.45
ILMN_1218981	Aldh1a7	Aldehyde Dehydrogenase 1 Family Member A1	−1.39
ILMN_1244513	Gbp3	Guanylate Binding Protein 3	−1.42
ILMN_2671165	Krt23	Keratin 23	−1.03
ILMN_2486906	Wisp2	WNT1 Inducible Signaling Pathway Protein 2	1.37
ILMN_3143404	Mup2	Major urinary protein 2	2.35
ILMN_1213817	Mup3	Major urinary protein 3	2.69
ILMN_3065459	Mup2	Major urinary protein 2	3.47
ILMN_2873822	Aebp1	AE Binding Protein 1	1.45
ILMN_2635229	Thbs2	Thrombospondin 2	1.59
ILMN_2875730	Mup1	Major urinary protein 1	2.95
ILMN_2659680	Serpina1b	Serpin Family A Member 1	1.94
ILMN_1215859	Serpina1b	Serpin Family A Member 1	1.96
ILMN_2953807	Mup6	Major urinary protein 6	2.72
ILMN_1225570	Serpina1d	Serpin Family A Member 1	1.99
ILMN_2443330	Ttr	Transthyretin	3.03
ILMN_1247156	Apoa2	Apolipoprotein A2	2.83
ILMN_1213954	Sgk1	Serum/Glucocorticoid Regulated Kinase 1	1.48
ILMN_2904137	Ambp	Alpha-1-Microglobulin/Bikunin Precursor	2.09
ILMN_2623393	Apoa1	Apolipoprotein A1	2.38
ILMN_2749037	Chchd10	Coiled-Coil-Helix-Coiled-Coil-Helix Domain Containing 10	1.92
ILMN_2753809	Mmp3	Matrix Metallopeptidase 3	1.37
ILMN_2993745	Ahsg	Alpha 2-HS Glycoprotein	1.91
ILMN_3158499	Mdk	Midkine	1.48
ILMN_2788223	Kng1	Kininogen 1	1.88
ILMN_2984332	Hal	Histidine Ammonia-Lyase	5.25

**Figure 3:**
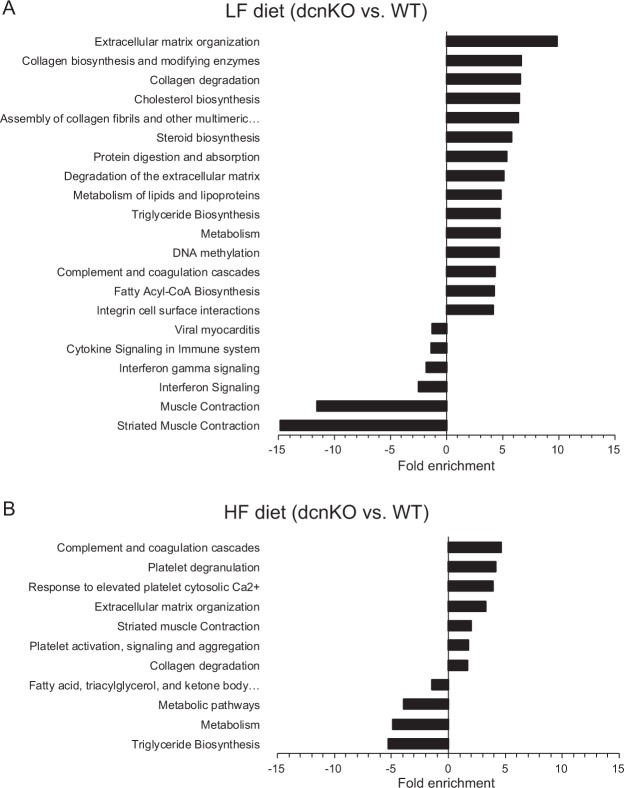
Enriched KEGG pathways for differentially expressed genes in iWAT comparing *Dcn*KO and WT C57BL/6J mice. Total RNA was purified from adipose tissue and subjected to microarray analysis. Genes with a false discovery rate (FDR) below 20% were analyzed in the Kyoto Encyclopedia of Genes and Genomes (KEGG) gene ontology database, and data are presented as log fold enrichment for each significantly enriched category (p < 0.05) for up- and down-regulated genes in *Dcn*KO vs. WT, respectively. iWAT, inguinal white adipose tissue; LF, low-fat diet; HF, high-fat diet; *Dcn*KO, decorin knock-out mice; WT, wild-type mice.

To more systematically assess biological functions represented by the top regulated genes, we performed pathway enrichment analysis based on the Kyoto Encyclopedia of Genes and Genomes (KEGG) database. Consistent with the role of decorin in extracellular matrix, transcripts induced in *Dcn*KO on LF were enriched in the pathways Extracellular matrix organization, Collagen biosynthesis and modifying enzymes, and Collagen degradation (Fig. 3A), and transcripts downregulated in *Dcn*KO mice were enriched in the pathways Striated muscle contraction and Muscle contraction (Fig. 3A). On the HF diet, upregulated transcripts in *Dcn*KO were enriched in the pathways, e.g. complement and coagulation cascades and platelet degranulation (Fig. 3B), while downregulated transcripts were enriched in the pathways, e.g., triglyceride biosynthesis and metabolism (Fig. 3B), supporting a role for decorin in adipose tissue lipid metabolism and inflammation.

### Adipose *DCN* expression increases upon profound fat loss in humans

**Figure 4:**
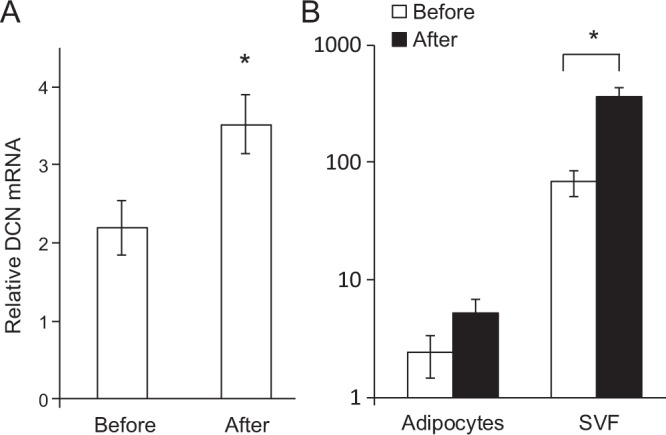
Adipose expression of *DCN* mRNA before and after bariatric surgery. Subcutaneous adipose tissue was collected from morbidly obese patients before and one year after bariatric surgery (gastric sleeve). RNA was purified, cDNA was synthesized and *DCN* mRNA was measured by qPCR and calculated relative to the reference gene *HPRT*, in whole tissue (**A**, n = 16) as well as in pairs of isolated adipocytes (n = 6) and stromal-vascular fraction (SVF, n = 6) (**B**). *p-value < 0.05.

Finally, we analyzed *DCN* mRNA expression by qPCR in subcutaneous adipose tissue of human subjects before and one year after bariatric surgery (biliopancreatic diversion with duodenal switch (n = 13)^32^. A significantly increased expression of decorin mRNA was observed one year after surgery (Fig. 4A). The results were confirmed in another set of patients with another surgical procedure (gastric sleeve), (n = 6) (Fig. 4B). Subcutaneous adipose tissue samples from these patients were fractionized into an adipocyte fraction and a stromal vascular fraction (SVF). Decorin was predominantly expressed in the SVF and the significant increase in expression one year after surgery is only seen in the SVF although the same tendency seems to be present in the adipocyte fraction (Fig. 4B).

## Discussion

We here studied the impact of loss of decorin on obesity-related phenotypes, hypothesizing that decorin plays an important role in the regulation of body weight, glucose tolerance and adipose tissue function. Our primary finding is that loss of decorin in mice impaired glucose tolerance and increased feed efficiency upon overfeeding, indicating that decorin plays a causal role in regulating glucose metabolism. We further mapped the global gene expression in adipose tissue associated with this phenotype, which pointed to reduced extracellular matrix organization and triglyceride biosynthesis in the *Dcn*KO mice exposed to HF diet, as well as increased complement and coagulation cascades. Our findings in patients showing increased decorin expression one year after bariatric surgery support the observation in the mice.

Insulin resistance associates with adipocyte hypertrophy in humans^38^. Interestingly, despite the impaired glucose tolerance and tendency of increased weight gain in the *Dcn*KO mice, we found no evidence of increased adipocyte size. This suggests that loss of decorin negatively affected glucose homeostasis independently of adipocyte hypertrophy. Such a dissociation of insulin resistance and adipocyte hypertrophy is consistent with that seen in collagen VI knockout mice, which show a marked hypertrophy of individual adipocytes concomitant with improved glucose tolerance^8^.

Our genome-wide gene expression data indicate that decorin exerts positive metabolic effects by different mechanisms. Firstly, several ECM-related genes were differentially regulated in adipose tissue of the *Dcn*KO mice, consistent with decorin serving as a component of the ECM in various tissues, including adipose tissue where it is highly expressed particularly in the visceral depot and in the stromal-vascular fraction^14,15^. Obesity is characterized by extensive reorganization of the extracellular matrix in adipose tissue^39^, and factors related to adipose ECM function have been implicated in obesity-related pathogenesis^40^ particularly linked to excess collagen fibril formation (fibrosis)^8,41^. Increased ECM deposition and rigidity in adipose tissue is thought to prevent adipose tissue expansion, while also increasing the risk of glucose intolerance during overfeeding due to ectopic lipid accumulation. Previous reports have shown increased expression of decorin along with several other ECM components in obesity^6,14,16^, and we also found increased decorin expression in both adipose tissue and muscle after overfeeding. However, our causal mouse data and prospective human data indicate that decorin does not promote adipose fibrosis and insulin resistance. To the contrary, the reduced glucose tolerance in the *Dcn*KO mice is consistent with previous observations that decorin inhibits TGFβ and connective tissue growth factor, two established pro-fibrotic factors^25,42^.

Further, in the knockout compared to wild-type mice we found up-regulation of adipose genes involved in complement and coagulation cascades, platelet degranulation and response to elevated platelet cytosolic Ca2+. Thrombin-induced increases in cytosolic Ca2+ promote platelet reactivity, a response that is augmented in insulin resistant cells because insulin signaling normally counteracts these pro-coagulation effects of thrombin^43,44^. Reducing thrombin action was found to directly improve insulin sensitivity in leptin resistant obese mice, suggesting a causal role for prothrombrotic processes in obesity-related insulin resistance^45^. Thus, an effect of decorin knockout on pro-coagulation cascades may have contributed to the increased diet-induced insulin resistance in these mice.

An isoform of decorin has been described as a receptor for the adipose-expressed factor resistin^18^ named for its ability to promote insulin resistance in animal models^46^. The resistin receptor is formed after cleavage of a glycanation site from full-length decorin, and was found to specifically present on the surface of adipose stromal cells^18^. It could be expected that loss of this decorin-derived resistin receptor improves glucose tolerance, but we rather found reduced glucose tolerance in the *Dcn*KO mice. In humans, resistin is primarily expressed in peripheral-blood mononuclear cells (PMBCs), which along with vascular cells show pro-inflammatory responses to resistin^47^. However, the importance of adipose resistin as a causal factor in insulin resistance remains unclear^47^.

**Figure 5:**
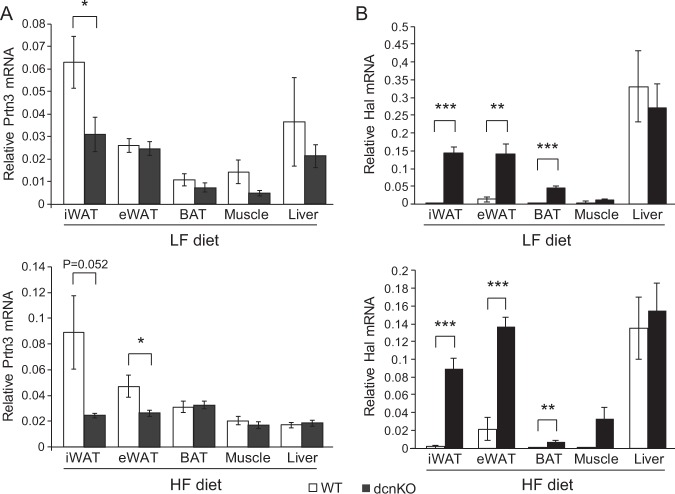
Tissue expression of the two most differentially expressed genes between *Dcn*KO and WT C57BL/6J mice. Global gene expression was measured in iWAT by Illumina microarrays, revealing *Prtn3* and *Hal* as the most down- and up-regulated genes, respectively, comparing dcnKO and WT mice. mRNA expression levels of *Prtn3* (**A**) and *Hal* (**B**) in different metabolic tissues were measured by qPCR and calculated relative to the reference gene *Rps13*. iWAT, inguinal white adipose tissue; eWAT, epididymal white adipose tissue; BAT, interscapular brown adipose tissue. *p-value < 0.05; **p-value < 0.01; *p-value < 0.001.

In our search for specific genes that might have mediated effects of decorin knockout on glucose tolerance via adipose tissue, we performed global gene expression profiling and identified two particularly regulated genes, *Prtn3* (downregulated in knockout mice) (Fig. 5A) and *Hal* (upregulated in knockout mice) (Fig. 5B). *Prtn3* encodes Proteinase 3 which degrades ECM components including elastin, fibronectin and several collagen subtypes^48^. Prtn3 is highly expressed in polymorphonuclear leukocytes such as neutrophils, and plays an important role in antimicrobial defense mechanisms. Prtn3 also plays a role in noninfectious inflammation^49^ and elastin-derived peptides accumulate with aging and directly promote insulin resistance^50^. Of note, a dense mesh of elastin fibers forms in visceral adipose tissue during development of obesity, while in subcutaneous adipose tissue the elastin fibers occur more linearly and colocalize with macrophages^51^. However, the downregulation of *Prtn3* does not readily explain the reduced glucose tolerance in the *Dcn*KO mice, based on the proposed stimulatory roles of *Prtn3* in inflammation and insulin resistance. More likely, the decreased glucose tolerance involved the most upregulated gene, *Hal*, which encodes a histidase that degrades the amino acid histidine. Histidine supplementation has been found to reduce oxidative stress, inflammation and fat mass and to improve glucose metabolism in mice, humans and pre-adipocyte cultures^37,52–54^. Thus, increased *Hal* expression in the *Dcn*KO mice may have contributed to the insulin resistant phenotype by increasing the degradation of histidine.

Decorin is expressed in several tissues and cell types, therefore we cannot say with certainty which tissues were primarily responsible for the metabolic phenotype of the knockout mice. We found that decorin mRNA expression increased upon overfeeding in white and brown adipose tissues as well as in skeletal muscle. The decorin expression was relatively low in liver where overfeeding did not notably affect decorin levels. Interestingly, we found that Uncoupling protein 1 (Ucp1) mRNA was downregulated in brown adipose tissue (BAT) of dcnKO mice on a HF diet, suggesting that decreased thermogenesis may have contributed to the phenotype of these mice (Fig. S2). No differences in mRNA expression of Pgc1a or Prdm1 were found (Fig. S2). To know the effect of decorin loss specifically in adipose tissue, we would need conditional knockout models targeting the most relevant stromal vascular cell types. This was beyond the scope of the present study.

In conclusion, we found that decorin plays a causal role in protecting against diet-induced hyperglycemia. Previous reports have showed elevated decorin gene expression coupled with increased circulating insulin levels and insulin resistance in rodents and humans^14,16^. In contrast we found that fasting blood glucose was increased in absence of *Dcn* in mice fed HF diet in addition to higher glucose levels throughout the GTT. Moreover, the upregulation of decorin in adipose tissue following profound fat loss in humans after bariatric surgery, which has a substantial effect on improving glucose homeostasis, further supports a role for decorin in maintaining glucose tolerance. Loss of decorin associated with adipose genes involved in complement and coagulation cascades, as well as elevated adipose expression of *Hal* which has been implicated in inflammation and glucose intolerance through degradation of histidine. Taken together, our data show that loss of decorin causes glucose intolerance upon overfeeding, at least in part via changes in adipose tissue function.

## Supplementary information

Supplemental
