# Supplementary material for: Absence of the proteoglycan decorin reduces glucose tolerance in overfed male mice
Source: Sci Rep. 2019 Mar 15;9:4614. doi: 10.1038/s41598-018-37501-x (PMC6420637; doi:10.1038/s41598-018-37501-x)
Supplement: Supplementary file 1 — Supplemental [file 41598_2018_37501_MOESM1_ESM.pdf]

Absence of the proteoglycan decorin reduces glucose tolerance in overfed male mice

Jessica Svärd[1,2], Therese H Røst[1,2,*], Camilla E N Sommervoll[1,2,*], Christine Haugen[1,2,], Oddrun A Gudbrandsen[3], Anne E Mellgren[3,4], Eyvind Rødahl[3,4], Johan Fernø[1,2], Simon N Dankel[1,2], Jørn V Sagen[1,2] and Gunnar Mellgren[1,2‡]

[1] KG Jebsen Center for Diabetes Research, Department of Clinical Science, University of Bergen, N-5020 Bergen, Norway,
[2] Hormone Laboratory, Haukeland University Hospital, N-5021 Bergen, Norway,
[3]Department of Clinical Medicine, University of Bergen, Norway,
[4]Department of Ophthalmology, Haukeland University Hospital, Bergen, Norway

[‡] Corresponding author: gunnar.mellgren@uib.no, [*]These authors contributed equally.

Suppl. Fig. 1

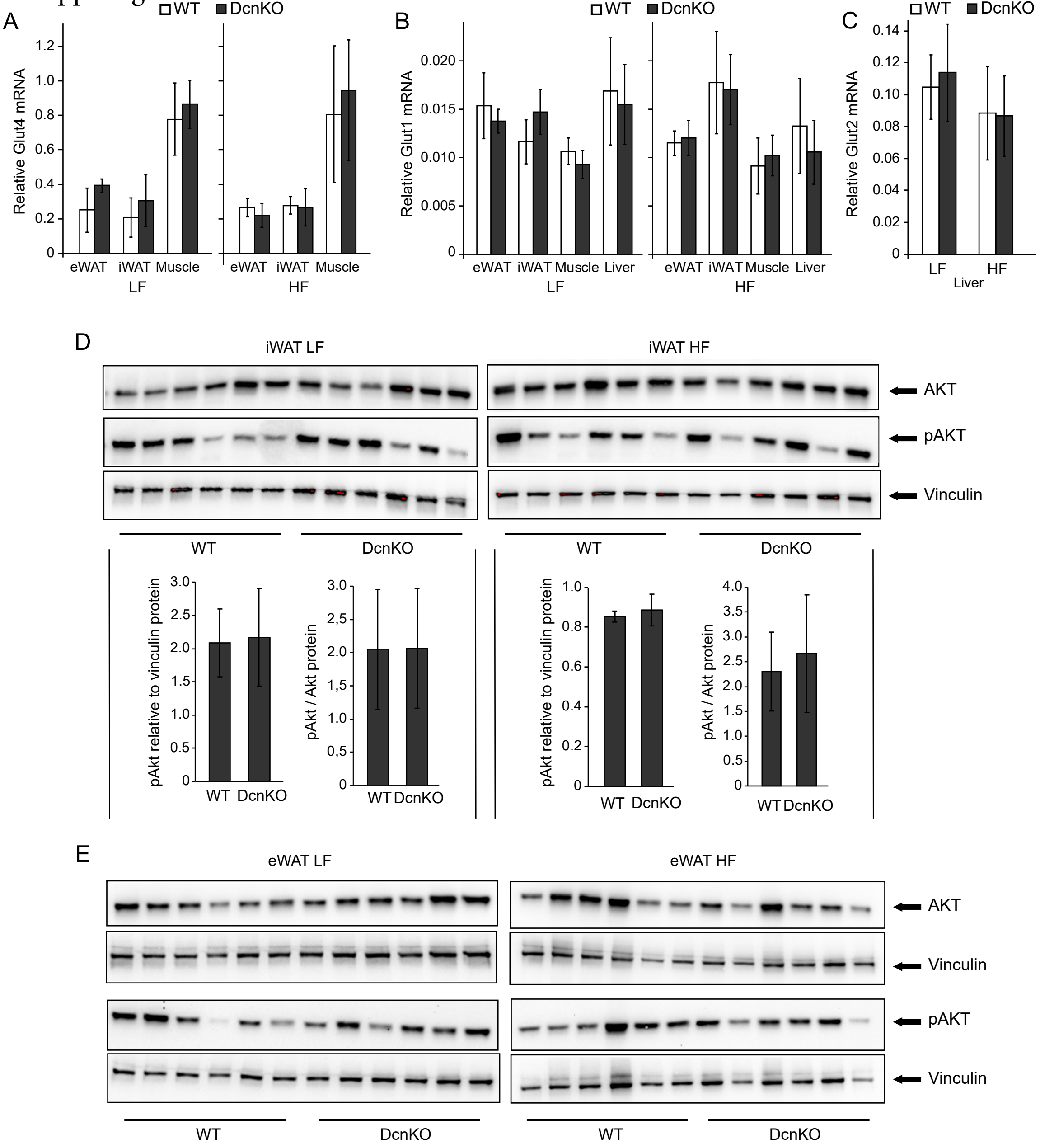

A
WT
DcnKO
Relative Glut4 mRNA
eWAT iWAT Muscle
LF
HF
B
Relative Glut1 mRNA
eWAT iWAT Muscle Liver
C
Relative Glut2 mRNA
LF HF
Liver
D
iWAT LF
iWAT HF
AKT
pAKT
Vinculin
WT
DcnKO
pAkt relative to vinculin protein
pAkt / Akt protein
E
eWAT LF
eWAT HF
AKT
Vinculin
pAKT
Vinculin
WT
DcnKO

Suppl. Fig. 2

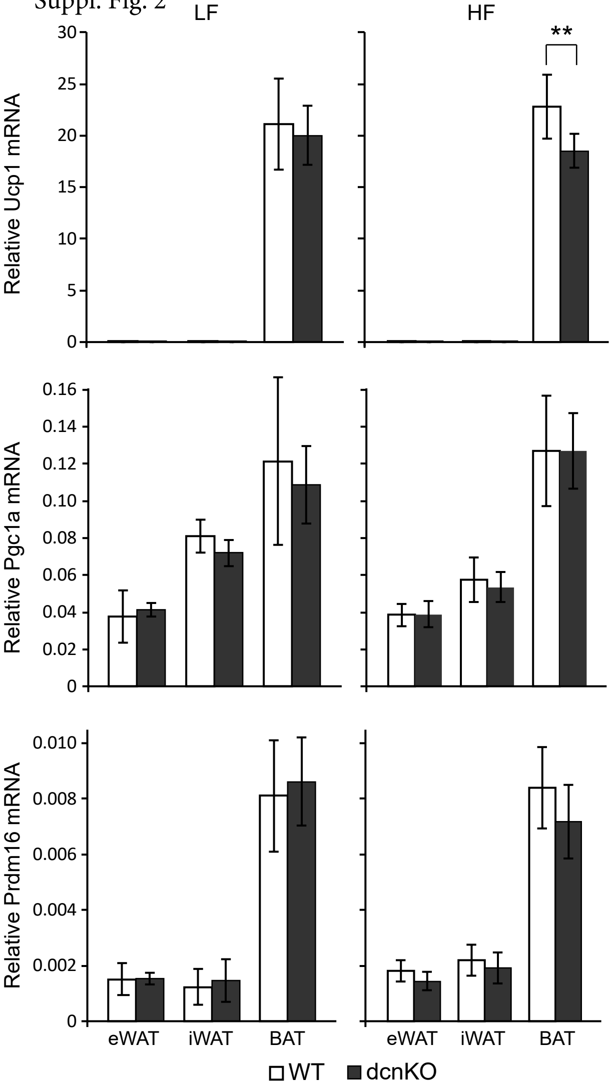
LF
HF
**
Relative Ucp1 mRNA
30
25
20
15
10
5
0
Relative Pgc1a mRNA
0.16
0.14
0.12
0.10
0.08
0.06
0.04
0.02
0
Relative Prdm16 mRNA
0.010
0.008
0.006
0.004
0.002
0
eWAT
iWAT
BAT
WT
dcnKO

Supplemental Figure Legends

Supplemental Figure 1. Gene expression of glucose transporters and protein levels of Akt pathway. *Glut4* mRNA in adipose tissue and skeletal muscle was measured by qPCR (A), *Glut1* mRNA in adipose tissue, skeletal muscle and liver was measured by qPCR (B) and *Glut2* mRNA in liver was measured by qPCR, all calculated relative to the reference gene *Rps13* (C). Protein levels of Akt pathway in iWAT and eWAT from WT and *Dcn*KO mice fed LF or HF diet.

Supplemental Figure 2. Adipose tissue expression of Ucp1, Pgc1a and Prdm16. mRNA expression levels of *Ucp1*, *Pgc1a and Prdm16* in different adipose tissues were measured by qPCR and calculated relative to the reference gene *Rps13*. iWAT, inguinal white adipose tissue; eWAT, epididymal white adipose tissue; BAT, interscapular brown adipose tissue. *, p-value < 0.05; **, p-value < 0.01;
